# Advances in the Diagnosis and Management of Psychotic Symptoms in Neurodegenerative Diseases: A Narrative Review

**DOI:** 10.1177/08919887231164357

**Published:** 2023-03-20

**Authors:** Andreea L. Seritan

**Affiliations:** 1 University of California, San Francisco 8785 Department of Psychiatry and UCSF Weill Institute for Neurosciences, CA, USA

**Keywords:** neurodegeneration, movement disorder, dementia, prodrome, review, management

## Abstract

**Background:** Approximately 15% of older adults may experience psychotic phenomena. Primary psychiatric disorders that manifest with psychosis (delusions, hallucinations, and disorganized thought or behavior) account for less than half. Up to 60% of late-life psychotic symptoms are due to systemic medical or neurological conditions, particularly neurodegenerative diseases. A thorough medical workup including laboratory tests, additional procedures if indicated, and neuroimaging studies is recommended. This narrative review summarizes current evidence regarding the epidemiology and phenomenology of psychotic symptoms encountered as part of the neurodegenerative disease continuum (including prodromal and manifest stages). **Results:** Prodromes are constellations of symptoms that precede the onset of overt neurodegenerative syndromes. Prodromal psychotic features, particularly delusions, have been associated with an increased likelihood of receiving a neurodegenerative disease diagnosis within several years. Prompt prodrome recognition is crucial for early intervention. The management of psychosis associated with neurodegenerative diseases includes behavioral and somatic strategies, although evidence is scarce and mostly limited to case reports, case series, or expert consensus guidelines, with few randomized controlled trials. **Conclusion:** The complexity of psychotic manifestations warrants management by interprofessional teams that provide coordinated, integrated care.

## Introduction

Approximately 15% of older adults may experience psychotic phenomena.^1^ The combined prevalence of the two most common primary psychotic disorders (schizophrenia and delusional disorder) and mood disorders with psychotic features in adults older than 65 is 5-6%, with secondary psychosis accounting for the remainder.^2,3^ It is estimated that about 60% of new-onset psychotic symptoms in late life reflect underlying systemic medical or neurological conditions, most often neurodegenerative diseases.^4^ In contrast to primary psychotic syndromes, visual hallucinations (VH) are more common than auditory hallucinations (AH) in neurodegenerative diseases, with a variable prevalence of delusions. When patients over 40 years old present with psychosis for the first time in their life, a thorough workup is recommended. Additionally, atypical onset ages (higher than the average onset age in the general population), an insidiously progressive course, unusual clinical presentations, and treatment resistance should prompt an assessment for neurodegenerative diseases.^5,6^

Over the past decade, a body of work has accumulated on the previously underrecognized neurodegenerative disease prodromes. Prodromes are constellations of symptoms that occur prior to the onset of overt neurological (e.g., cognitive or motor) features and often include psychiatric manifestations. This review will cover neurodegenerative disease prodromes (also referred to as preclinical, premanifest, or premotor), as well as overt (also termed manifest or clinical) stages. Of note, in the Alzheimer’s disease (AD) literature, mild cognitive impairment (MCI) and a newer construct, mild behavioral impairment (MBI) that describes patients with behavioral symptoms who may or may not have cognitive deficits, are classified as prodromal.^7^ In contrast, the earlier stage (corresponding to normal cognition, subjective cognitive decline, and MBI-preclinical) is considered preclinical.^7^ For the purposes of this review, prodromes include *all* symptoms that precede the overt neurological symptoms of neurodegenerative diseases, focusing on psychiatric aspects. Another important distinction is that between neurodegenerative diseases and major neurocognitive disorders (NCDs). Although many patients with neurodegenerative diseases ultimately develop major NCDs, not all do. The focus of this review is on neurodegenerative diseases, not limited to major NCDs.

The purpose of this narrative review was to identify and highlight studies published in the last decade focusing on the recognition, diagnosis, and management of psychotic symptoms associated with the neurodegenerative disease continuum (including prodromal and manifest stages).

## Methods

A first PubMed literature search was conducted using the terms (*Alzheimer’s disease* or a*myotrophic lateral sclerosis* or *dementia* or *dementia with Lewy bodies* or *fragile X-associated tremor/ataxia syndrome* or *frontotemporal dementia* or *Huntington’s disease* or *Parkinson’s disease* or *spinocerebellar ataxia*) and (*psychosis* or *psychotic* or *delusions* or *hallucinations*). A second search was then performed using the same terms and adding (*prodrome* or *prodromal*). A third search was conducted including each of the neurodegenerative diseases listed above and the term *antipsychotic.* In the next step, each antipsychotic medication (*aripiprazole*, *brexpiprazole*, *cariprazine*, *clozapine*, *iloperidone, lurasidone, olanzapine*, *pimavanserin*, *quetiapine*, *risperidone, paliperidone*) was used as a search term paired with each neurodegenerative disease, to identify any treatment studies that may have been missed. Additional articles were retrieved by examining the reference lists of the studies identified through the above search strategy, as well as textbook chapters. Case reports, case series, review articles, treatment guidelines, and clinical trials, including randomized clinical trials (RCTs) published in English between 2012 and 2022 were included. Primary sources were preferred over review articles, if both were available on a given topic. Articles published before 2012 were retained if newer evidence was not available or if they were “classics”, such as the Clinical Antipsychotic Trials of Intervention Effectiveness-Alzheimer’s Disease (CATIE-AD) study.^8^ Publications referring only to the cognitive, autonomic, or other non-behavioral aspects of neurodegenerative diseases and those not meeting the definition of prodrome were excluded.

**Figure 1.**
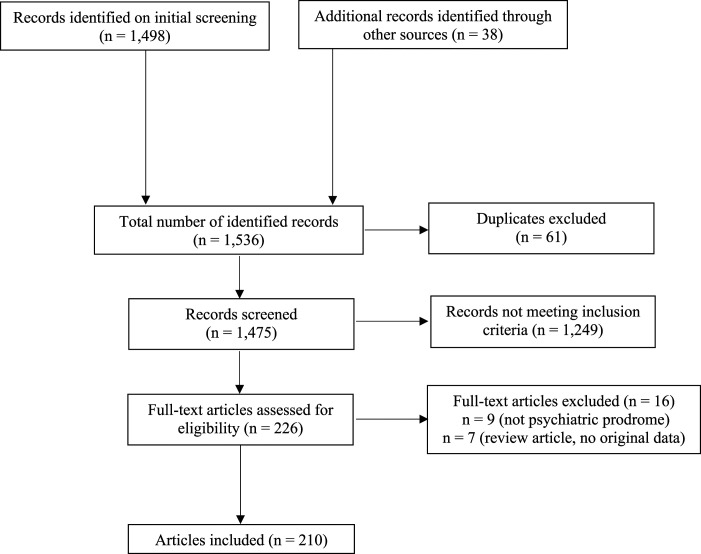
PRISMA flow chart of literature search and article selection.

For the workup section, an outline of possible medical etiologies was developed based on our clinical experience and a previous comprehensive review article,^4^ followed by targeted PubMed searches to identify publications that included older adults (for example, using the terms, *neurosyphilis* and *psychosis* and *older*). Figure 1 depicts the PRISMA flow diagram for literature search and article selection.^9^

## Results

### Diagnostic Workup

The first step to elucidate the origin of new acute-onset psychotic symptoms in older adults is to seek underlying reversible causes, if any, and rule out delirium. A thorough workup, especially searching for infectious etiologies such as urinary tract infection (UTI), pneumonia, and viral encephalitis will help uncover systemic medical conditions that can cause acute mental status changes. It is also important to inquire about any recent medication changes (prescribed and over the counter), as well as alcohol and drug use. In particular, agents with anticholinergic properties, benzodiazepines, and opioids can contribute to delirium.^10,11^

Delirium occurs in up to 50% of hospitalized older adults and can persist for weeks after the underlying cause is treated.^12^ Risk factors include age over 75 years; cognitive impairment, especially major NCD; prior history of delirium; history of cerebrovascular accident or transient ischemic attack; functional, visual, or hearing impairment; comorbidity or severity of medical illness; depression; and alcohol use disorders.^12^ Delirium manifests with attention fluctuations and sleep-wake cycle disturbances; disorientation is not as common as previously thought.^13^ Hallucinations (more frequently visual than auditory) can occur in 40-70% of patients, while delusions (often persecutory) have been described in 25-79% of individuals who experience delirium.^4,13^

Once delirium is ruled out, the workup of late-life psychotic symptoms includes routine blood tests (complete blood count, electrolytes, glucose, and hepatic and renal function tests), thyroid stimulating hormone, vitamin B_12_, and urine drug screen.^4,14^ Additional laboratory tests include erythrocyte sedimentation rate, folate, heavy metals, rapid plasma reagin, and human immunodeficiency virus and severe acute respiratory syndrome coronavirus-2 (SARS-CoV-2) tests.^14^ Lumbar puncture can help elucidate an infectious etiology of meningitis or encephalitis and support a diagnosis of multiple sclerosis or autoimmune encephalitis, although the latter two conditions seldom present de novo in older adults.^15^ Electroencephalograms can aid in diagnosing seizure disorders, which can also be associated with psychosis.^16^ Neuropsychological testing is helpful in differentiating depression from major NCDs and identifying patterns of deficits that may indicate specific neurodegenerative diseases. For example, cognitive deficits outlining a hippocampal pattern (poor recall, flat learning curve, and poor recognition) along with language and visuospatial skills impairment are pathognomonic for AD.^17,18^ Patients with AD forget new information rapidly and do not benefit from cues; list-learning tasks are most sensitive for detecting the memory deficits in early AD.^19^ Patients with the behavioral variant of frontotemporal dementia (bvFTD) or frontal AD variant (also known as behavioral dysexecutive AD variant) show profound executive dysfunction.^17,18,20^ Individuals with movement disorders such as Parkinson’s disease (PD) or Huntington’s disease (HD) demonstrate a frontal-subcortical deficit pattern, with executive dysfunction and poor spontaneous recall, but generally preserved learning and intact recognition (cues help).^17,21^ Mixed major NCDs such as dementia with Lewy bodies (DLB) and fragile X-associated tremor/ataxia syndrome (FXTAS) combine hippocampal and frontal deficit patterns.^18,22,23^

Depression is often associated with neurodegenerative diseases such as AD, PD, and HD. In a study of adults aged 60-89 evaluated on 5 cognitive domains (verbal episodic memory, executive function, processing speed, constructional praxis, and language/semantic memory), patients with late-life depression scored below the 5th percentile in at least two domains, while people with mild AD had lower scores in 3 or more domains.^24^ Patients with depression also tend to give more “I don’t know” answers and show impaired performance on measures of learning and free recall as well as tests that rely on attention, effort, and speed.^25^ Older adults with depression perform generally better than people with AD and demonstrate retrieval difficulties on memory testing, as opposed to amnestic deficits, typical of AD.^25^ In contrast, the neuropsychological profile in PD is quite similar to the one found in depression, so it is harder to differentiate.^25^ Neuropsychological evaluation results should be interpreted in clinical context.

Finally, neuroimaging studies, especially brain magnetic resonance imaging (MRI), can corroborate clinical findings and support the diagnosis.^26,27^ Brain MRIs are particularly helpful when evaluating patients with gradual cognitive decline, acute-onset confusion, or behavioral changes and are best to identify subcortical pathology, white matter, and microvascular ischemic changes.^19,26^ Head computerized tomography (CT) scans help diagnose intracranial hemorrhage, acute cerebrovascular accidents (ischemic or hemorrhagic), mass-occupying lesions, normal pressure hydrocephalus, and reveal cortical atrophy with resulting ventricular enlargement.^19^ Functional imaging studies such as positron emission tomography (PET) and single photon emission computed tomography (SPECT) can detect neuronal metabolic abnormalities before changes are visible on structural imaging investigations such as CT or MRI, although they are not readily available at all hospitals.^27^ Patterns of cortical atrophy or hypometabolism identified through neuroimaging can help distinguish neurodegenerative diseases, although findings should always be interpreted in clinical context. A posterior (predominantly parietal and hippocampal) configuration can be found in AD, whereas an anterior (frontal and/or anterior temporal) pattern is common in bvFTD.^20,26^

Table 1 highlights laboratory tests and procedures that can uncover potentially reversible etiologies of late-life psychotic symptoms.

**Table 1.** Suggested Workup for New-Onset Psychotic Symptoms in Older Adults.^14,28-35^

Test or procedure	Clinical significance of abnormal results
Laboratory test	
Blood urea nitrogen, creatinine	Renal disease
Complete blood count	Infection
	Lymphoproliferative disorders
Electrolytes	Hyponatremia/hypernatremia
	Hypokalemia (e.g., Cushing syndrome)
Glucose	Hypoglycemia/hyperglycemia
Glycated hemoglobin (HbA_1c_)	Diabetes mellitus
Liver function tests	Hepatic disease
Ammonia	Hepatic encephalopathy
Antinuclear antibody	Systemic lupus erythematosus
	Rheumatologic disease
Thyroid function tests^a^	Hypothyroidism/hyperthyroidism
Urinalysis	Urinary tract infection
Urine drug screen	Substance intoxication/withdrawal
Vitamin B_12_	Vitamin B_12_ deficiency
Folate	Folate deficiency
Vitamin D	Vitamin D deficiency
Erythrocyte sedimentation rate	Inflammation
	Vasculitis
	Temporal arteritis
SARS-CoV-2 tests	COVID-19
Calcium^b^	Hypocalcemia/hypercalcemia (e.g., hyperparathyroidism)
Heavy metals	Intoxication
HIV	HIV-associated neurocognitive disease
Rapid plasma reagin; fluorescein treponema antibody	Neurosyphilis
Procedures	
Chest radiography	Pneumonia
Lumbar puncture	Autoimmune encephalitis
	Meningitis
	Multiple sclerosis
	Viral encephalitis
Electroencephalogram	Creutzfeldt-Jakob disease
	HSV encephalitis
	Seizures
Head computerized tomography	Acute cerebrovascular accident
	Mass-occupying lesions (e.g., tumor, infection)
	Subdural hematoma
Brain magnetic resonance imaging	Autoimmune encephalitis
	CNS vasculitis
	Microvascular ischemic changes
	Multiple sclerosis
	Normal pressure hydrocephalus
	Neurodegenerative disease

CNS = central nervous system; COVID-19 = coronavirus disease 2019; HIV = human immunodeficiency virus; HSV = herpes simplex virus; SARS-CoV-2 = severe acute respiratory syndrome coronavirus-2.

^a^5-15 of patients with severe hypothyroidism may develop psychosis.

^b^Both severe hypo- and hypercalcemia can be associated with psychosis.

Table 2 summarizes systemic medical diseases, substances, and medications that may be associated with psychotic symptoms in older adults.

**Table 2.** Systemic Medical Diseases, Substances, and Medications Associated with Psychotic Symptoms in Older Adults.^4,11,17,25,28-30,32-42^

Etiology	Examples
Substance intoxication	Alcohol (alcoholic hallucinosis)
	Cannabis
	Cocaine
	Hallucinogens (ketamine, LSD, mushrooms, PCP, psilocybin)
	Methamphetamine
	MDMA
Substance withdrawal	Alcohol
	Sedative, hypnotic, anxiolytic (e.g., benzodiazepine)
	Opioids
Medications	Anticholinergic and antihistaminic agents (benztropine, cimetidine, diphenhydramine, hydroxyzine)
	Antiparkinsonian agents (e.g., amantadine, bromocriptine, levodopa, pramipexole, ropinirole, rotigotine, trihexyphenidyl)
	Antibiotic-associated encephalopathy (quinolones, macrolides, procaine penicillin)
	Anticonvulsants (levetiracetam, zonisamide)
	Corticosteroids
	Digoxin
	Interferon
	Opioids
	Psychostimulants (e.g., amphetamine, dextroamphetamine, lisdexamfetamine, methylphenidate)
Metabolic and endocrine conditions	Acute intermittent porphyria
	Adrenal disease
	Electrolyte imbalances (e.g., sodium, potassium, calcium)
	Heavy metals (e.g., lead, mercury)
	Hepatic failure
	Hypoglycemia/hyperglycemia
	Hypoparathyroidism/hyperparathyroidism
	Hypothyroidism/hyperthyroidism; Hashimoto’s thyroiditis
	Renal failure
	Vitamin deficiencies (B_12_, D, folate)
Neurological conditions	Alzheimer’s disease
	Amyotrophic lateral sclerosis
	Autoimmune encephalitis
	Brain tumors
	CADASIL
	Cerebral vasculitis
	Cerebrovascular accidents (basal ganglia, cerebellar, frontal, temporal, or parietal lobe)
	COVID-19
	Dementia with Lewy bodies
	Frontotemporal dementia
	HIV infection
	Huntington’s disease
	Hypoxia
	Meningitis
	Multiple sclerosis
	Neurosyphilis
	Paraneoplastic syndromes
	Parkinson’s disease
	Prion disease (e.g., Creutzfekdt-Jakob)
	Seizure disorders
	Subdural hematoma
	Systemic lupus erythematosus
	Traumatic brain injury
	Viral encephalitis (HHV-6, HSV)

AH = auditory hallucinations; CADASIL = cerebral autosomal dominant arteriopathy with subcortical infarcts and leukoencephalopathy; COVID-19 = coronavirus-19 disease; HHV-6 = human herpesvirus-6; HIV = human immunodeficiency virus; HSV = herpes simplex virus; LSD = lysergic acid diethylamide; MDMA = 3,4-methylenedioxy-methamphetamine; PCP = phencyclidine.

Though infrequently used in clinical practice, genetic testing can identify mutations linked to neurodegenerative diseases associated with psychosis.^27^ For example, hexanucleotide repeat expansions caused by *chromosome 9 open reading frame 72 (C9orf72)* mutations are the most common genetic abnormality in behavioral variant frontotemporal dementia (bvFTD) and familial amyotrophic lateral sclerosis (ALS) and are associated with an increased risk for psychosis.^43,44^ A thorough interview, inquiring about family history of neurodegenerative diseases, is also important to identify genetic causes.^27^

Table 3 highlights the most common neurodegenerative diseases caused by nucleotide repeat expansions.

**Table 3.** Neurodegenerative Diseases Caused by Nucleotide Repeat Expansions.^44-50^

Disease	Transmission	Chr.	Gene	Protein	Nucleotide repeat
Hexanucleotide					
FTD/ALS; bvFTD	Autosomal dominant	9	*C9orf72*	*C9orf72* protein	GGGGCC
Trinucleotide					
FXTAS	X-linked	X	*FMR1*	*FMRP*	CGG
Friedreich’s ataxia	Autosomal recessive	9	FXN	Frataxin	GAA
Huntington’s disease	Autosomal dominant	4	HTT	Huntingtin	CAG
Spinocerebellar ataxias					
➢ Type 1	Autosomal dominant	6	ATXN1	Ataxin-1	CAG
➢ Type 2	Autosomal dominant	12	ATXN2	Ataxin-2	CAG
➢ Type 3 (Machado-Joseph disease)	Autosomal dominant	14	ATXN2	Ataxin-3	CAG

ALS = amyotrophic lateral sclerosis; bvFTD = behavioral variant frontotemporal dementia; chr. = chromosome; *C9orf72* = *chromosome 9 open reading frame 72; FMR1* = fragile X mental retardation 1; *FMRP* = fragile X mental retardation protein; FXTAS = fragile X-associated tremor/ataxia syndrome.

### Psychotic Symptoms in Manifest Neurodegenerative Diseases

Psychotic symptoms are fairly common in manifest neurodegenerative syndromes and portend a poor prognosis, being associated with cognitive and functional decline and earlier death.^51-55^

**AD** is the most common neurodegenerative disease and major NCD worldwide, accounting for over 60% of cases.^56,57^ Psychotic symptoms typically occur in the middle stage and resolve in advanced disease (or are no longer communicated as patients lose verbal abilities).^51,57^ A large study of National Alzheimer’s Coordinating Center (NACC) data also identified delusions in 12% and hallucinations in 3% of people with mild AD.^58^ Overall, delusions (mainly paranoid, of theft, or misidentification) range in prevalence from 31% to 59% across studies.^57,59-61^ Reeves and colleagues^60^ posited that persecutory delusions appear earlier in the course of AD and are associated with dysfunction in frontostriatal circuits, whereas misidentification delusions emerge later and reflect limbic system changes. Hallucinations can occur in 16-41% of patients; simple VH predominate, although AH can also be present.^3,57,62^ In a sample of 1,808 adults with AD and other major NCDs characterized neuropathologically, hallucinations were more common in patients with mixed AD/Lewy body disease pathology than in those with AD or Lewy body disease alone.^63^

Presence of psychotic symptoms can skew clinical diagnosis. In a study of approximately 1,000 patients with pathologically-confirmed AD, patients with psychosis were five times more likely to be misdiagnosed with DLB, whereas those without psychosis were more likely to receive a false-positive diagnosis of AD, when in fact they had vascular lesions.^64^

Psychotic symptoms occur late in the course of **PD** and may arise in cognitively intact people, not just those with major NCD.^65,66^ Well-formed VH are typical, with a lifetime prevalence approaching 60%; AH are less frequent and can occur in up to 20% of people with PD.^52,66,67^ Delusions (mainly of infidelity, paranoid, or of misidentification) have been described in 5-10% of patients.^66-68^ Minor psychotic phenomena include auditory or visual illusions, passage hallucinations (brief visions of a person or animal passing in the periphery of the visual field), and extracampine hallucinations (sense of presence), which can manifest in half of patients.^67,69^ Psychosis may also be caused or exacerbated by treatment with levodopa or dopamine agonists,^66^ in which case delusions can take on a grandiose quality (e.g., having superpowers) and be associated with elevated mood, poor sleep, dopamine dysregulation syndrome, and/or impulse control disorders.

**DLB** is the third most common neurodegenerative disease in the U.S., after AD and PD.^56^ The major NCD occurs prior to or within 1 year of parkinsonism onset, although not all patients will develop parkinsonism.^70^ Recurrent, well-formed VH are present early and constitute a core clinical feature; hallucinations in other modalities and systematized delusions can also occur and are considered supportive features.^70^ Almost half of patients can harbor delusions, and up to 80% display VH.^61,70-72^ Delusions of misidentification, including Capgras syndrome (the false belief that a familiar person, often a close family member or caregiver, has been replaced by an identical-looking impostor) are frequent.^61,73,74^ Approximately 36% of patients with DLB had AH in a recent study, and 61% exhibited VH; AH often accompanied VH, like a “soundtrack”.^75^ Table 4 highlights the clinical features of DLB, updated by the DLB Consortium in 2017.^70^

**Table 4.** Clinical Features of Dementia with Lewy Bodies.^70^

*Essential feature:* dementia that occurs before or concurrently (within 1 year) with parkinsonism.
*Core clinical features* (the first 3 typically occur early and persist throughout the disease course):
1. Fluctuating cognition with pronounced variations in attention and alertness
2. Recurrent visual hallucinations, typically well-formed and detailed
3. REM sleep behavior disorder, which may precede cognitive decline
4. One or more spontaneous cardinal features of parkinsonism: bradykinesia, rest tremor, or rigidity
*Supportive clinical features:*
1. Severe sensitivity to antipsychotic agents
2. Postural instability
3. Repeated falls
4. Syncope or other transient episodes of unresponsiveness
5. Severe autonomic dysfunction, e.g., constipation, orthostatic hypotension, urinary incontinence
6. Hypersomnia
7. Hyposmia
8. Hallucinations in other modalities
9. Systematized delusions
10. Apathy, anxiety, and depression
DLB is less likely:
• If another systemic medical or neurological illness (e.g., cerebrovascular disease) is present
• If parkinsonian features are the only core clinical feature and appear for the first time at a stage of severe dementia.

DLB = dementia with Lewy bodies; REM = rapid eye movement.

**FTD** is the second most common form of early-onset major NCD.^27^ FTD is divided into two main clinical syndromes: bvFTD and primary progressive aphasia (PPA), further subdivided into nonfluent variant (nfvPPA) and semantic variant (svPPA).^76^ The newly described logopenic variant of PPA (lvPPA) is associated with AD pathology in over 80% of cases and is considered an AD variant.^77^ Behavioral manifestations are most salient in bvFTD and include apathy, loss of empathy, disinhibition, hyperorality, and compulsive behaviors, along with anxiety, depression, and executive dysfunction.^20^ A parallel classification describes frontotemporal lobar degeneration (FTLD) as a group of clinically and neuropathologically distinct syndromes which includes the FTD syndromes, progressive supranuclear palsy (PSP), corticobasal degeneration (CBD), and bvFTD with motor neuron disease (FTD-MND), with the exemplar FTD-ALS. The FTLD subtypes and their corresponding genetic and neuropathological underpinnings known to date are detailed in an excellent review by Elahi and Miller.^18^ Neuropathologically, the two most common FTLD subtypes are FTLD-tau (characterized by misfolded tau aggregate inclusions) and FTLD-TDP (resulting from the accumulation of 43-kDa transactive response DNA binding protein, TDP-43).^18^ In a series of 97 patients with autopsy-confirmed FTLD, nearly one third experienced psychotic symptoms including paranoid ideation, delusions, or hallucinations (mainly visual).^78^ Gossink et al.^79^ prospectively followed 22 patients with probable or definite bvFTD for 2 years; 5 (23%) developed delusions, hallucinations, or suspiciousness.

Although usually associated with slower disease progression, *C9orf72* mutations confer an increased risk of psychosis in both the prodromal and manifest FTD stages.^43,44,59,77,80-82^ Moreover, psychosis can be the presenting symptom.^43,44^ In a cohort of 398 individuals with bvFTD, nfvPPA, svPPA or an overlap of these syndromes, 32 patients were found to display *C9orf72* mutations, of whom 12 (32%) presented with frank psychotic symptoms (primarily delusions), and 9 others exhibited bizarre behavior.^44^ Delusions can be persecutory, somatic, grandiose or of infidelity, whereas hallucinations include all modalities.^81,82^ Benussi and colleagues^83^ explored the progression of neuropsychiatric symptoms (NPS) over a decade in a large cohort comprised of 232 patients with bvFTD, half of whom harbored *C9orf72* mutations. The prevalence of hallucinations increased from 10% shortly after diagnosis, to 23% 2 years later. Delusions were also common, ranging from 7% at diagnosis to 16% after 2 years.^83^ However, there was significant sample attrition over time and few mutation carriers were seen after the 2-year follow-up point; for this reason, later data should be interpreted with caution.

Neuropathologically, *C9orf72* mutations align with the FTLD-TDP subtype.^18^ Naasan et al.^74^ conducted a retrospective chart review of 372 people with pathologically characterized neurodegenerative syndromes, of whom 111 (27%) had endorsed psychotic symptoms. Patients with FTLD-TDP pathology were significantly more likely to have delusions, particularly in the first 3 years of the disease, when compared to the AD and FTLD-tau groups. Patients with FTLD-TDP were also more likely to display paranoid ideation and grandiose or erotomanic delusions compared to those with AD or FTLD-tau changes.^74^

Psychosis is exceedingly rare in PSP and CBD syndromes, although case reports have been published.^59,84^

Up to 22% of patients with **ALS** meet FTD criteria; about half remain cognitively intact, and the remainder develop milder deficits and personality changes.^85,86^ NPS include apathy (which can be present in up to 80% of patients), depression, anxiety, disinhibition, psychosis, and stereotyped behavior.^85,87-90^ Psychotic symptoms are infrequent (1-2%) in patients with ALS without FTD.^88^

**HD** is an autosomal dominant, fully penetrant neurodegenerative disease associated with > 36 CAG repeats, with onset usually in mid-adulthood.^91^ Psychiatric manifestations include apathy, depression, agitation/aggression, anxiety, obsessive-compulsive features, mania, and psychosis.^92-94^ The prevalence of psychotic manifestations (predominantly delusions) in HD had been previously estimated at 3-12%.^92,95^ However, two recent large cohort studies found rates of 13-18%.^54,96^ The latter two studies did not differentiate hallucinations from delusions; hence, more granular prevalence data are not available. Psychotic symptoms can range from poorly systematized paranoid ideation and isolated delusions to florid schizophrenia-like psychosis.^97^

**Spinocerebellar ataxias (SCAs)** are autosomal dominant, adult-onset neurodegenerative diseases of the cerebellum and spinal cord, although onset age can range from childhood to late life.^98,99^ There are over 40 SCA types currently known, the most common being SCA3 (also known as Machado-Joseph disease). SCAs often present with pure neurological phenotypes, although several subtypes can also be associated with cognitive impairment and psychiatric symptoms.^98^ In addition to psychosis, neuropsychiatric features include depression, anxiety, aggression, and personality changes.^100-103^ The prevalence of psychotic symptoms is difficult to establish, in part because of the heterogeneity of this group of diseases. Case reports and series have depicted individuals with SCA type 1, 2, 3, 6, 7, 10, 14, and 17 who exhibited delusions or hallucinations.^101-106^ The largest study involved 112 patients with Machado-Joseph disease, of whom 5 (4%) reported psychotic symptoms; all had hallucinations (either auditory or visual), along with delusional or disorganized thinking.^103^ Albeit rare, SCA17 involves the basal ganglia and has a clinical picture that mimics HD (chorea with cerebellar signs); psychotic symptoms may be more frequent.^99,105^

Psychosis is also infrequent in **FXTAS**, a neurodegenerative disease that affects older carriers of fragile X mental retardation (*FMR1*) gene premutations, with 55-200 CGG repeats.^45^ FXTAS occurs in approximately 40-75% of male premutation carriers (with penetrance increasing with age) and 16-20% of female carriers and is characterized by intention tremor, cerebellar ataxia, parkinsonism, cognitive decline, and NPS.^45,107,108^ Neuropsychiatric features include anxiety, depression, disinhibition, impulsivity, mood lability, irritability, apathy, and agitation.^107-111^ In a study of 55 carriers with FXTAS, 5 (9%) met the Diagnostic and Statistical Manual (DSM) IV-TR criteria for psychotic disorders, reporting paranoid delusions or hallucinations (visual or olfactory).^112^ Accelerated cognitive decline with or without VH has infrequently been described in individuals with gray zone alleles (45-54 CGG repeats) or in rare cases of FXTAS co-occurrence with other neurodegenerative processes, such as AD.^113,114^

Table 5 summarizes the clinical features of common neurodegenerative diseases that can present with psychosis during the manifest stage.

**Table 5.** Clinical Features of Neurodegenerative Diseases Associated with Psychosis.^45,50,66,70,71,75,79-81,92,96,98,99,103,107,112,115-117^

Neurodegenerative disease	AD	DLB	bvFTD	FXTAS	HD	PD	SCA
Typical onset age	> 65 y.o.^a^	65-85 y.o.	45-65 y.o.	> 50 y.o.	40 y.o.^b^	> 65 y.o.^c^	35-50 y.o.^d^
Sex ratio	F:M = 3:2	M:F = 2:1	M > F	M >> F	M = F	M:F = 3:2	M = F
Core clinical features	Early memory & learning deficits	Fluctuating cognition	Early decline in personal & social conduct	Cerebellar ataxia	Choreoathetosis	Bradykinesia	Cerebellar ataxia
	Language deficits	Recurrent, vivid VH	Apathy	Intention tremor	Dystonia (esp. juvenile form)	Resting tremor or rigidity	Pyramidal signs
	Visuo-spatial deficits	Parkinsonism	Poor insight	Parkinsonism	Bradykinesia	Postural instability Falls	Dysarthria
		REM sleep behavior disorder	Stereotyped/ compulsive behaviors				Nystagmus
Other clinical features	Executive dysfunction (in frontal variant)	Neuroleptic sensitivity	Executive dysfunction	Primary ovarian insufficiency (F)	Apathy	Dystonia	Ophthalmoplegia
			Carbohydrate craving	Migraines	Depression	Dyskinesia	Extrapyramidal signs
				Thyroid disease	Suicidality	Autonomic dysfunction	Seizures
Psychotic symptoms							
Delusions	9-59%	50%	2-23% (57% in *C9orf72* mutation carriers)	5%^e^	12%^f^	7-10%	5%^g^
Hallucinations^h^	16% (6-41%)	80%	18% (36% in *C9orf72* mutation carriers)	3%	2%	53-60%	5%^g^
• Auditory	8-12%	36%	9-10%	─	─	22%	Case reports
• Visual	7-23%	60-80%	7-9%	1%	2%	16-38%	Case reports

AD = Alzheimer’s disease; DLB = dementia with Lewy bodies; bvFTD = behavioral variant frontotemporal dementia; FXTAS = fragile X-associated tremor/ataxia syndrome; HD = Huntington’s disease; PD = Parkinson’s disease; SCA = spinocerebellar ataxia; F = female; M = male; VH = visual hallucinations; REM = rapid eye movement; *C9orf72 = chromosome 9 open reading frame 72*.

^a^5-6% of patients have early onset AD (before age 65).

^b^5-10% of patients develop a juvenile HD form, with onset before age 20.

^c^3-5% of patients have young onset PD (before age 50); there is a very rare juvenile form, with onset before age 21.

^d^Onset ages vary for different SCA types and can range from 3 to 75 years old.

^e^This estimate is based on a single study; results need to be replicated.

^f^Newer studies in larger cohorts found 13-18% prevalence for psychosis (hallucinations not separated from delusions).

^g^Prevalence data based on a single study of SCA3 (Machado-Joseph disease).

^h^Includes auditory, visual, olfactory, and minor phenomena, where present.

### Psychotic Symptoms as Part of Neurodegenerative Disease Prodromes

This section covers common psychiatric prodromes of neurodegenerative diseases. It is important to note that subtle cognitive deficits can also occur in preclinical stages; however, a comprehensive review of cognitive prodromal manifestations is beyond the scope of this review. Several neurodegenerative disease prodromes are well characterized. For example, anosmia, constipation, and rapid eye movement (REM) sleep behavior disorder are part of a well-known prodrome encountered in synucleinopathies such as PD, DLB, and, less often, multiple system atrophy.^118-124^ To date, prodromes with psychiatric symptoms have been described in AD,^125-132^ ALS,^88,89,133-135^ bvFTD,^5,136-139^ DLB,^121,124,126,127,140-145^ FXTAS,^146^ HD,^91,93,96,147,148^ PD,^6,118-120,123^ and SCAs.^101,149^ The most common prodromal NPS are apathy, anxiety, depression, and REM sleep behavior disorder. Albeit less frequent, psychosis can also occur in AD, ALS, bvFTD (especially among *C9orf72* mutation carriers), DLB, and HD prodromes.^93,96,125,128,133-138,143,145,148^

The MBI construct, initially proposed by Taragano et al.^139^ as a prodrome of FTD, was later expanded to describe an at-risk stage for all major NCDs. MBI has been extensively studied in recent years as part the AD prodrome, along with MCI. MBI is subdivided into 5 domains: reduced motivation/apathy, affective dysregulation, impulse dyscontrol, social inappropriateness, and abnormal perception or thought content (e.g., delusions, hallucinations) and can co-occur with or even precede MCI.^150^ MBI prevalence varies depending on participant age, setting, and instrument used, ranging from 3.5% among outpatients aged ≥ 50 seen in a psychiatry clinic^151^ to 14% of patients with MCI in a primary care practice^152^ and 34% in a sample of 1,377 community-dwelling adults aged 72-79 years (49% in the MCI subgroup among them).^153^ Patients with MBI were found to have a higher risk (hazard ratio, HR, 8.07) of converting to major NCD (mainly AD) over a follow-up period of up to 104 months compared to those with MCI without MBI (HR 7.05).^151^ The most common MBI domain was affective dysregulation (64%), followed by abnormal perception or thought content (21%).^151^ In a recent web-based longitudinal study of 8,181 older adults (median age, 63 years), 11% of women and 14% of men met MBI criteria.^154^ All MBI domains were associated with cognitive decline; the association with psychosis had the largest effect size, but only in men, underscoring the importance of exploring sex differences.^154^

In the NACC database analysis conducted by Apostolova et al., 3% of participants with amnestic MCI (often an AD precursor) and 4% of those with nonamnestic MCI had delusions, while 1% in each group exhibited hallucinations.^58^ Ismail et al.^155^ reported a 3.1-10.5% pooled prevalence of delusions and 1.3-2.6% frequency of hallucinations among individuals with MCI.

The prodrome that is perhaps best known to neurologists and psychiatrists is that of **DLB**, consisting of anxiety, apathy, depression, or REM sleep behavior disorder.^121,122,124,126,127,140-144^ Psychotic symptoms are also prominent.^126,140,143,145^ In a large cohort study including 148 patients with prodromal DLB, 64% had hallucinations and 13% displayed delusions.^140^ Utsumi and colleagues^145^ followed longitudinally 21 patients who presented with recurrent catatonia, delusions, or hallucinations and were diagnosed with DLB a decade later, on average. Wyman-Chick et al.^143^ conducted a retrospective chart review of 116 NACC Uniform Data Set database participants who did not have a major NCD diagnosis initially and converted to DLB at a subsequent visit. The authors investigated NPS prevalence for up to 5 years prior to diagnosis. They found that 19% of participants had hallucinations and 14% endorsed delusions 4 years prior to DLB diagnosis, with VH incidence rising to 20-25% 2 years later.^143^

Anxiety, apathy, depression, and irritability are common NPS which can occur as part of the **AD** prodrome, along with psychosis.^58,129-132,156^ Presence of psychotic symptoms increases risk for AD.^125,128,156^ In a large Italian nested case-control cohort study, medical records of 1,889 primary care patients who developed AD were examined retrospectively for 10 years prior to diagnosis.^128^ Patients with a history of hallucinations had a four-times higher likelihood of developing AD; anxiety, agitation, aberrant motor behavior, memory deficits, and depression also increased risk.^128^ In a Japanese study of 234 individuals with MCI (mean age, 73 years) followed for up to 3 years, 3% had psychotic symptoms.^156^ Delusions were the only factor significantly associated with conversion to AD (unadjusted HR, 2.9).^156^ Apolipoprotein E ε4 carriers with NPS have an even higher risk of progressing to AD.^125,155^ In an analysis of 11,453 cognitively intact NACC database participants, ε4 carriers who experienced delusions or hallucinations had a significantly higher chance of developing AD within several years compared to those without NPS; specifically, presence of delusions amplified the risk thirteen-fold.^125^

Among all neurodegenerative diseases, **bvFTD** is the best mimic of psychiatric disorders. One third to half of patients are initially diagnosed with psychiatric conditions, and the bvFTD diagnosis can be delayed by 5-6 years.^27,136,137^ In a cross-sectional study based on standardized interviews with the caregivers of 46 people with bvFTD, 37% of patients had received a diagnosis of bipolar disorder (most often), schizophrenia, or schizoaffective disorder.^137^ The association of FTD with ALS is also salient. Lillo et al.^133^ examined a series of 18 patients with **FTD-ALS** who presented with behavioral changes. Patients with concurrent onset of behavioral and motor symptoms and those initially diagnosed with ALS who developed behavioral changes later were excluded. Nine (50%) of the 18 patients had delusions and 5 (28%) endorsed VH.^133^ Delusions were persecutory, of theft, and erotomanic in nature. Presence of delusions predicted a subsequent diagnosis of ALS.^133^

Turner et al.^134^ found that hospital admissions for diagnoses of schizophrenia, bipolar disorder, depression, or anxiety were significantly associated with a new diagnosis of **ALS** in the following year. The association remained significant only for depression hospitalizations up to 5 years preceding ALS diagnosis. Similarly, Longinetti et al.^135^ identified a significant association of depression, neurotic (anxiety, obsessive-compulsive, dissociative, and somatoform) disorders, and drug abuse/dependence, as well as other neurodegenerative diseases (FD, AD, PD, or other dementia) with risk of developing ALS. This association was strongest in the year preceding the ALS diagnosis but extended for the previous 2-5 years.^135^

Psychosis is also part of the **HD** prodrome. In the European Registry of HD mutation carriers, 48% of participants had a motor onset, 20% first experienced psychiatric symptoms, and 8% had initial cognitive deficits, whereas 13% had a mixed presentation.^148^ Almost 40% had a lifetime history of severe psychiatric disease, including psychosis, aggression, and suicidality.^148^ In another large cohort study using the Enroll-HD international database, 3% of individuals with pre-manifest HD reported psychosis.^96^ Other neuropsychiatric features of prodromal HD are apathy, depression, irritability, and anxiety.^93^

Prodromal psychiatric manifestations are rare in **SCAs**, besides REM sleep behavior disturbances.^149^ A case report depicted one man initially diagnosed with schizophrenia at age 22, who had persistent psychotic symptoms, progressively declined over time, and was ultimately found to have SCA2 ten years later.^101^

Minor hallucinations can predate the onset of motor symptoms in **PD**.^157^ A recent study also found an association between late-life psychosis and probability of having prodromal PD, calculated based on depression, constipation, and subthreshold parkinsonism.^158^

Table 6 summarizes psychiatric prodromes of neurodegenerative diseases known to date.

**Table 6.** Psychiatric prodromes of neurodegenerative diseases.^6,88,89,91,93,96,101,118-135,137-149^

Neurodegenerative disease	Psychiatric prodrome
Alzheimer’s disease	Anxiety, apathy, depression, irritability, psychosis (especially in apoE ε4 carriers)
Amyotrophic lateral sclerosis	Anxiety, mood disorders (depression, bipolar), psychosis, substance use
Behavioral variant frontotemporal dementia	Anxiety, depression, psychosis (especially in *C9orf72* carriers)
Dementia with Lewy bodies	Anxiety, apathy, depression, REM sleep behavior disorder, psychosis
FXTAS	Anxiety, depression
Huntington’s disease	Apathy, anxiety, depression, psychosis, aggression, suicidality
Parkinson’s disease	Anxiety, depression, REM sleep behavior disorder
Spinocerebellar ataxia	Psychosis (rare), REM sleep behavior disorder

ApoE = apolipoprotein E; *C9orf72* = *chromosome 9 open reading frame 72*; FXTAS = fragile X-associated tremor/ataxia syndrome; REM = rapid eye movement.

### Diagnostic Criteria

#### Psychosis in Manifest Neurodegenerative Diseases

Jeste and Finkel^57^ were the first to attempt to differentiate the psychotic symptoms associated with AD from those occurring in late-life primary psychotic disorders. They formulated separate diagnostic criteria for psychosis of AD.^57^ Later, a National Institute of Neurological Disorders and Stroke – National Institute of Mental Health work group developed PD psychosis criteria.^159^ Recently, an International Psychogeriatric Association (IPA) expert panel proposed clinical and research criteria for psychosis in major, as well as mild NCD, taking into consideration the fact that psychotic symptoms can occur earlier in the course of NCDs.^160^ The International Society to Advance Alzheimer’s Research and Treatment (ISTAART) Professional Interest Area psychosis subgroup led by Fischer et al.^7^ reviewed the previous criteria sets for psychosis of AD and other major NCDs, including those by Jeste and Finkel,^57^ Lyketsos et al.,^161^ and DSM-5. In revising the AD psychosis criteria, Fischer and colleagues^7^ advanced a new major NCD framework that includes neuroimaging and other biomarkers alongside clinical elements, thus mirroring current diagnostic criteria for other neurodegenerative diseases such as DLB and PSP.^70,162^ Of note, all the diagnostic criteria mentioned above, except for the ones regarding PD psychosis, imply the presence or later development of a major NCD.

#### Neurodegenerative Disease Prodromes

Research criteria have been proposed for prodromal PD and DLB.^120,124,163^ Psychiatric symptoms (depression ± anxiety) are included in the PD prodrome, although the emphasis is on other clinical aspects and biomarkers.^120^ The DLB prodrome also includes clinical features and evidence-based biomarkers.^124^ However, given the prominent psychiatric symptoms, the DLB prodrome has been classified in 3 subtypes: DLB-MCI, delirium onset-DLB (with provoked or spontaneous delirium), and psychiatric disorder-DLB (manifested primarily as late-onset mood or psychotic disorder).^124,163^ These presentations differ markedly from MBI symptoms and may be severe enough to require hospitalization.^124^

ISTAART introduced diagnostic criteria for MBI in 2016.^150^ MBI is defined as changes in behavior or personality reflected in the aforementioned 5 domains (reduced motivation, affective dysregulation, impulse dyscontrol, social inappropriateness, and abnormal perception or thought content) that start at or after age 50, are observed by patients, informants, or clinicians, and persist at least intermittently for 6 months or longer.^150^ Symptoms have to be of sufficient severity to cause impairment in interpersonal, social, or occupational functioning. Having a primary psychiatric disorder or a major NCD are exclusionary criteria. MBI can be diagnosed concurrently with MCI, but co-occurrence is not necessary. A 34-item MBI-Checklist was also developed and validated to aid in clinical diagnosis and research studies.^164,165^

### Management of Psychotic Symptoms in Neurodegenerative Diseases

Behavioral strategies are always recommended as first step in the management of NPS in neurodegenerative diseases. The American Psychiatric Association Guidelines for treatment of psychosis and agitation in AD suggest using antipsychotic medications only after behavioral approaches have failed, and if there is risk of self-harm or harm to others.^166^ An international panel of experts recommended the DICE (Describe, Investigate, Create, and Evaluate)^167^ model and music therapy to address overall NPS, agitation, and psychosis.^168^ No RCTs of behavioral interventions for psychosis associated with neurodegenerative diseases have been conducted to date. Diederich et al.^169^ studied 46 patients with PD and classified the strategies they used to cope with VH into 3 categories: visual (focusing better on the hallucinatory object, looking away from it, or focusing on another object), cognitive (turning the light on or telling themselves these phenomena are not real and will resolve shortly), and interactive (discussing with family and caregivers for reality testing and reassurance). Regardless of cognitive status, patients used cognitive strategies most often (69%), followed by interactive (62%), then visual (33%) techniques.^169^

There are few RCTs for the pharmacological management of psychosis in major NCDs, and even fewer published in the last decade.^170,171^ Most treatment recommendations are based on anecdotal evidence and expert consensus.^166,168,170-172^ A recent network meta-analysis suggested that aripiprazole might be the most effective and safe antipsychotic for treating the behavioral and psychological symptoms of major NCDs; however, psychosis outcomes were not analyzed separately.^173^ Of note, all antipsychotics have a boxed warning regarding the increased risk of death for older adults with major NCD-related psychosis.^174^ There are no data on the newer antipsychotics brexpiprazole, cariprazine, iloperidone, or lurasidone.

#### Alzheimer’s Disease

The CATIE-AD study examined the effectiveness and safety of second-generation antipsychotics for AD-associated agitation or psychosis. In this multi-site, double blind RCT, 421 outpatients received olanzapine (mean dose, 5.5 mg/day), quetiapine (mean dose, 56.5 mg/day), risperidone (mean dose, 1 mg/day), or placebo.^8^ There were no differences across groups regarding effectiveness or time to discontinuation,^8^ but there was a modest advantage for olanzapine and risperidone in improving NPS.^175^ In an earlier systematic review by Sink et al.,^176^ olanzapine 5-10 mg/day and risperidone 1mg/day also showed significant, but modest effects in reducing delusions, hallucinations, and aggression associated with AD or vascular major NCD.

Aripiprazole, a partial D_2_ receptor agonist, showed benefit in an RCT of 487 nursing home residents with AD psychosis who were randomized to placebo or aripiprazole 2, 5 or 10 mg/day. Aripiprazole 10 mg daily dose resulted in a significant improvement in the Neuropsychiatric Inventory-Nursing Home (NPI-NH) Psychosis subscale at ten weeks, in contrast to placebo.^177^

There have been few newer RCTs for treatment of psychosis in AD. The expert panel listed risperidone as first line and pimavanserin, discussed in detail below, and citalopram as promising alternatives.^168^ A phase II RCT including 181 nursing home residents with psychosis associated with AD showed benefit for pimavanserin 34 mg/day over placebo at 6 weeks, but differences were not maintained at 12 weeks.^178^ In a phase III trial that was stopped early for efficacy, patients with psychosis related to AD, PD, DLB, FTD, or vascular major NCD received open-label pimavanserin 34 mg daily for 12 weeks.^179^ Half of the responders were assigned to continue pimavanserin, and half to placebo. Responders had a lower risk of psychosis relapse if they continued pimavanserin, as opposed to stopping it.^179^

The Citalopram for Agitation in Alzheimer's Disease (CitAD) trial demonstrated a significant improvement in agitation associated with AD with citalopram up to 30 mg daily.^180^ A secondary analysis also found a reduction in delusions and hallucinations, with the best response noted in patients with concurrent agitation and psychosis.^181^ Of note, the maximum FDA-recommended citalopram daily dose for adults over age 60 is 20 mg.^182^

A 12-week double-blind RCT of low-dose lithium (150-600 mg daily) for agitation in AD was negative with regard to the primary outcome.^183^ Exploratory analyses yielded a statistically significant superior improvement in delusions, but not hallucinations, with lithium vs. placebo.

Secondary analyses of data from cholinesterase inhibitors (ChEIs) trials in AD have revealed potential benefit or reduced emergence of psychosis, although there is no RCT demonstrating specific benefit of ChEIs in the management of AD psychosis.^171^ In a study of over 17,000 individuals without prior psychotropic use from the Swedish Dementia Registry Study, use of ChEIs, particularly at higher doses, was associated with a lower likelihood of starting treatment with antipsychotics in patients with AD, but not DLB.^184^

#### Parkinson’s Disease

Since psychotic symptoms can be induced or exacerbated by dopaminergic medications, an important first step is to attempt to reduce these agents, as tolerated without worsening of motor symptoms. This should be done in collaboration with treating neurologists. Experts recommend reducing or stopping PD medications in the following order: anticholinergic agents, amantadine, dopamine agonists, monoamine oxidase inhibitors, catechol-O-methyltransferase inhibitors, and lastly, levodopa.^185^ If this strategy is ineffective, second-generation antipsychotics (never first-generation) can be used cautiously.

A recent systematic review and meta-analysis summarized all RCTs of antipsychotics used for PD psychosis.^186^ There have been no new RCTs since 2012, with the exception of the pimavanserin trials described below. Pimavanserin is the only FDA-approved agent for psychosis in a neurodegenerative disease (specifically, for delusions and hallucinations of PD) and was recommended by an expert panel as first line for PD psychosis.^172^ Pimavanserin has a novel mechanism of action, acting as a selective serotonin 5HT2A receptor inverse agonist. Cummings et al.^187^ reported significant improvements in psychotic symptom scores in a phase III 6-week long RCT comprising 199 adults (mean age, 72.4 years) with PD psychosis. This trial was followed by a 4-week open label extension, during which all participants received daily pimavanserin 34 mg and the Scale for the Assessment of Positive Symptoms for Parkinson's Disease Psychosis (SAPS-PD) scores continued to drop.^188^ Almost half of patients experienced side effects, leading to study discontinuation in 5.9% of cases.^188^ Pimavanserin takes 10-12 days to reach steady state and may need up to 6 weeks to achieve effect; the experts recommended discontinuing it after 8 weeks, if no benefit is noted.^172^ Further safety data, particularly in older adults, need to be collected. In 2018, the Food and Drug Administration did not find any new or unexpected safety risks in an analysis of all postmarketing reports of death and serious adverse events reported.^189^ However, in a retrospective cohort study of adults aged 65 or older with PD (of whom 2,186 were taking pimavanserin and 18,212 were not taking it), pimavanserin users had a significantly higher risk of hospitalization at 30 days and higher risk of death for up to 1 year after starting treatment, compared to non-users.^190^

Clozapine and quetiapine have low D2 receptor binding affinity; as such, they are least likely to cause motor symptom worsening. Clozapine is the only agent with sufficient evidence, based on two earlier RCTs and one meta-analysis that supported its efficacy in improving PD psychosis, with questionable benefit for motor symptoms.^172,186,191^ Clozapine requires frequent monitoring and can potentially have severe adverse effects such as agranulocytosis, albeit rare.^191^

Quetiapine is preferred in practice, although there is mixed evidence for its use.^186,191,192^ In a chart review of 5,297 Veterans Affairs patients with PD psychosis, quetiapine accounted for two thirds of antipsychotic prescriptions.^191^ Horn et al.^193^ conducted a retrospective study comparing pimavanserin to quetiapine for psychosis in patients 41-97 years old (mean age, 73 ± 8 years) with PD or DLB. Patients in the pimavanserin group were more likely to have a diagnosis of DLB and to have tried an antipsychotic medication previously. Pimavanserin was more often discontinued due to refractory psychosis (i.e., lack of efficacy), while quetiapine was more likely to be discontinued due to side effects.^193^

Evidence regarding the efficacy of ziprasidone in psychosis or acute agitation associated with PD is mixed, consisting mostly of case reports and a case series.^194,195^ Although generally well tolerated in PD, ziprasidone caused worsening of motor symptoms in patients with DLB. A 4-week, randomized, single-blind, open-label study including 14 patients compared ziprasidone (final mean dose, 35 mg/day) to clozapine (final mean dose, 32 mg/day) and found ziprasidone to be at least as effective as clozapine for PD psychosis.^195^

In a recent open-label trial, aripiprazole at 3 mg/day appeared effective, however 8 of the 24 participants reported worsening of motor symptoms.^196^ Olanzapine, risperidone, and aripiprazole should be avoided, based on evidence to date and expert recommendations.^172,186^

The ChEIs donepezil, galantamine, and rivastigmine have also been used for PD psychosis. However, the evidence is scarce and the results, unsatisfactory. Studies performed prior to 2012 were summarized in a Cochrane database systematic review.^197^ Of note, most studies were designed to evaluate cognitive functioning in patients with major NCD as primary outcome, with psychotic symptoms as secondary outcomes. There are few studies in cognitively intact people with PD and psychosis.^198^ One recent RCT randomized patients with PD and minor VH to rivastigmine 3 or 6 mg twice daily or placebo but was stopped early due to slow recruitment.^199^ The study was not adequately powered to evaluate primary outcomes, but the authors reported no group differences in progression to frank psychosis or major NCD during the 2-year follow up period.^199^

Anecdotal reports describe the benefits of antidepressants (citalopram, escitalopram, clomipramine, mianserin, mirtazapine, and venlafaxine) for the treatment of psychotic symptoms in patients with PD with or without comorbid depression, including one case report of a 67-year-old man whose VH were refractory to quetiapine, then clozapine, but improved with mirtazapine 30 mg at bedtime.^200^ The evidence regarding antidepressant use for PD psychosis is weak, limited to case reports or series.^200-203^

Electroconvulsive therapy (ECT) was shown to improve depression, psychosis, and motor symptoms in patients with PD, although the motor improvement was short-lasting.^204,205^

#### Dementia with Lewy Bodies

Patients with DLB are highly sensitive to antipsychotic parkinsonian side effects and may experience motor symptom exacerbation.^70,172^ Severe sensitivity to neuroleptics is a supportive clinical feature for a probable DLB diagnosis.^70^ For this reason, less potent D_2_ receptor blockers like quetiapine or clozapine are preferred. Quetiapine is favored in practice, although there is insufficient evidence for its efficacy.^193,206,207^ Several case reports have shown mixed results for clozapine, but trial data are lacking.^206-208^ Due to its novel mechanism of action, pimavanserin shows promise, yet its efficacy has not been proven to date.^193^ Similar to PD, olanzapine and risperidone can cause motor worsening and have unclear benefit, so they are best avoided.^206,207^ One case report revealed benefit of low-dose aripiprazole without exacerbation of parkinsonism but more data are needed.^72^

ChEIs have also been used for treating psychosis in mild to moderate DLB. The evidence is stronger than in PD. In an RCT of 140 patients with DLB randomized to receive donepezil 3, 5 or 10 mg daily or placebo for 12 weeks, there was significant improvement in hallucinations and delusions with donepezil 5 and 10 mg doses.^209^ The benefit was maintained over a 52-week open label phase.^210^ The same group conducted a 16-week phase III trial, followed by a 36-week open label arm using donepezil 10 mg daily.^211^ Among the 100 patients who completed the study, there was no difference compared to placebo.^211^ In a small study including 8 patients with DLB and VH, donepezil 10 mg daily was effective in treating symptom recurrence after initial resolution with 5 mg daily.^212^

ECT has also been tried for patients with DLB, but benefits are less robust compared to PD.^204,213^

#### Behavioral Variant Frontotemporal Dementia

Studies suggest a dopaminergic deficit in bvFTD.^214^ As such, quetiapine or aripiprazole may be preferred.^215,216^ There have been several case reports and case series using olanzapine, quetiapine, and risperidone for psychosis in bvFTD, but no RCTs.^82,86,217,218^ Clozapine 400 mg daily was helpful for a 26-year-old man with an initial diagnosis of schizoaffective disorder (later changed to FTD) with treatment-refractory psychosis and severe aggression.^219^ As discussed above, there is insufficient evidence for pimavanserin.^179^ The cholinergic system is only mildly affected in FTD.^18,214^ As such, ChEIs are not beneficial for cognition and may actually worsen behavioral symptoms.^86,216^

#### Huntington’s Disease

There are no RCTs of antipsychotics in HD; most treatment recommendations come from anecdotal experience and expert consensus.^50,220^ In practice, strong D_2_ blockers like haloperidol, risperidone, or olanzapine or the partial D_2_ agonist aripiprazole have been used to manage both chorea and psychosis.^148,221-223^ Aripiprazole is also recommended for treating psychosis with prominent negative symptoms.^50^ Less potent D_2_ blockers such as quetiapine and clozapine can also be helpful. Quetiapine showed positive results in case reports and a series comprised of 5 patients,^97,223,224^ whereas clozapine was found to be effective in two middle-aged adults with treatment-refractory HD psychosis.^225,226^ The International Guidelines for the Treatment of Huntington’s Disease suggest clozapine as first line for psychosis in patients with akinetic HD and disabling parkinsonian symptoms.^220^ HD psychosis can be severe and relentless; in some cases, combinations of two antipsychotics are necessary, although this should be avoided as much as possible due to risk of tardive dyskinesia and worsening parkinsonism, which often emerges later in the course of the disease.^223,227^

ECT has been used for medication-refractory cases, with mixed results.^220,225^

#### Spinocerebellar Ataxias

Given the low frequency of psychotic symptoms in SCAs, there is scarce evidence on the use of antipsychotics. Okamoto and colleagues^106^ described a 43-year-old man with a 15-year history of SCA3, who was admitted for depression and somatic and grandiose delusions. His depression and grandiosity responded to escitalopram 10 mg and olanzapine 2.5 mg daily; however, the somatic delusions persisted.^106^ Two other patients in the case series by Turk et al.^105^ were treated with olanzapine, and one with risperidone (doses not mentioned). Wexler and Fogel described a 37-year-old man with SCA type 10 and new-onset psychosis and violent behavior, whose symptoms responded to risperidone 2 mg twice a day.^102^

There are anecdotal reports on the use of quetiapine for psychosis in ***FXTAS***, given that parkinsonian features may be part of the clinical picture.^228^ Larger studies are needed. Table 7 summarizes the evidence discussed above.

**Table 7.** Antipsychotic Medications Used for Treatment of Psychotic Symptoms in Neurodegenerative Diseases.^8,72,82,92,97,168,179,187-189,192,194,207,217-219,221-225,229,230^

Medication (starting/target daily dose)	Strength of evidence/Other information
Alzheimer’s disease	
Risperidone 0.25 mg/1 mg	Good RCTs; modest effect in CATIE-AD
Olanzapine 2.5 mg/5-10 mg	Few RCTs; modest effect in CATIE-AD
Quetiapine 12.5 mg/200 mg	Negative RCTs; positive small case series
Aripiprazole 2 mg/10 mg	One positive RCT in nursing home patients
Pimavanserin 34 mg/34 mg	Insufficient evidence
Parkinson’s disease	
Clozapine 6.25 mg/50-75 mg	Few early RCTs; maximum dose reported 225 mg daily
Quetiapine 12.5 mg/125-225 mg	Mixed evidence; maximum dose reported 400 mg daily
Ziprasidone 20 mg/40 mg	Case reports/series, one open-label randomized trial
Pimavanserin 34 mg/34 mg	RCTs; mixed long-term and safety data to date
Dementia with Lewy bodies	
Quetiapine 12.5 mg/175 mg	1 RCT, 1 case series; mixed results
Clozapine 6.25 mg/50 mg	No studies
Aripiprazole 1.5 mg/3 mg	Case report
Pimavanserin 34 mg/34 mg	Insufficient evidence
BvFTD	
Risperidone 1 mg/6 mg	Case reports
Olanzapine 2.5mg/10 mg	Case series
Quetiapine 25 mg/150-800 mg	Case reports
Clozapine 12.5 mg/400 mg	Case report
Pimavanserin 34 mg/34 mg	Insufficient evidence
Huntington’s disease	
Risperidone 0.5-1 mg/3-6 mg	Case reports
Olanzapine 2.5 mg/10-20 mg	Case reports
Aripiprazole 2.5 mg/10-30 mg	Case reports
Quetiapine 25 mg/300-600 mg	Case reports; case series
Clozapine 12.5 mg/450-500 mg	Case reports

CATIE-AD = Clinical Antipsychotic Trials of Intervention Effectiveness–Alzheimer’s Disease; RCT = randomized controlled trial; bvFTD = behavioral variant frontotemporal dementia.

## Discussion

Psychotic symptoms associated with neurodegenerative diseases are multifaceted and can occur as part of both prodromal and manifest disease stages. Psychiatric prodromes constitute a newer area of interest, which has yet to be fully elucidated. Knowledge is evolving, and studies of increasing quality have been published over the last decade. This narrative review summarized the extant body of knowledge. The current terminology is heterogenous. Most authors agree on the term “prodrome”, while others used the terms “preclinical”, “premanifest”, “premotor”, or “predementia”. The ISTAART AD psychosis criteria distinguish the “preclinical” from the “prodromal” stage (the latter encompasses MBI and MCI).^7^

The neurodegenerative diseases included in this review were selected based on their prevalence in the general population (in descending order): AD, PD, DLB, FTD, HD, ALS, and SCAs.^56^ Conditions that have a described psychiatric prodrome, such as FXTAS, were also of interest. The strength of the evidence varied. Studies ranged from case reports,^101,102,106,121,138,141^ case series,^5,79,82,91,122,133^ retrospective chart or database reviews,^58,96,128,133-136,140,143,146-148^ and cross-sectional analyses^137^ to prospective longitudinal studies.^83,145^ Including case reports diluted the strength of the evidence summarized; however, a higher level of evidence, particularly with regard to treatment, is lacking at this time for less common neurodegenerative diseases (e.g., HD, SCAs, even bvFTD – see Table 7).

Psychiatric prodromes can predate overt neurodegenerative disease manifestations by 1-5 years in AD and ALS, 5-10 years in DLB, and two-to-three decades in bvFTD, FXTAS, HD, PD, and certain SCAs.^6,93,96,101,119-124,134,137,138^ This is clinically relevant, because patients may present with psychiatric symptoms long before they develop full-blown neurological syndromes. Hence, accurate recognition of psychiatric prodromes is paramount. Hallucinations are more common in the DLB prodromal stage,^140,143^ whereas delusions are more frequent during the AD and bvFTD prodromes.^58,125,133,154-156^ Of note, prodromal psychotic symptoms, particularly delusions, have been associated with an increased likelihood of receiving a neurodegenerative disease diagnosis within several years.^125,133,154,156^ In PD, delusions are considered to represent a deterioration of hallucinations, as distorted thought processes crystallize around misperceptions.^66^ As such, delusions may indicate more advanced disease, being succeeded by overt cognitive or motor manifestations shortly thereafter.

Accurate identification of the neurodegenerative disease prodromes will also allow early intervention with disease-modifying treatments, as these become available. The ISTAART MBI criteria^150^ are an excellent, albeit not disease-specific, starting point for a broader definition of major NCD prodromes. Nevertheless, the MBI syndrome does not entirely overlap with neurodegenerative disease prodromes. In the AD literature (and, to some extent, other major NCDs), the prodromal stage only includes MCI and MBI (much closer in time to the onset of clinically relevant cognitive deficits), while earlier manifestations are deemed preclinical.^7^ However, in the movement disorders literature (PD, DLB, ALS), prodromes are a broader concept, extending decades before motor symptoms occur and including autonomic and other clinical features, along with neuroimaging, genetic, and other biomarkers.^120,124,163^ While MBI is considered a pre-dementia stage, not all patients with neurodegenerative diseases and psychiatric prodromes (e.g., ALS, SCAs) will develop major NCDs.^85,98^ The MBI onset age is ≥ 50 years, yet psychiatric prodromal symptoms can occur in people as young as their 20’s.^6,101,137,138,146^ Lastly, presence of a psychiatric disorder precludes an MBI diagnosis.^150^ However, the majority of the studies focusing on prodromes reviewed here indicate that patients are often diagnosed with various psychiatric disorders prior to receiving a neurodegenerative disease diagnosis. These distinctions will be important to keep in mind as research and clinical knowledge advance and the neuropsychiatric field moves toward a unified prodrome definition and nomenclature. One possible expanded prodrome definition involves the emergence of NPS that precede the cognitive or motor manifestations of neurodegenerative diseases, as early as several decades in advance. Further research is needed to operationalize and validate a broader prodrome construct.

Mild psychotic symptoms can occur in cognitively intact, community-dwelling older adults.^7,155^ Two recent studies revealed a prevalence of 0.7-0.8% for delusions and 0.2-0.3% for hallucinations among cognitively intact older adults.^153,231^ These may represent prodromal symptoms that have not yet reached clinical significance, although late- or very late-onset primary psychotic disorders should enter the differential as well.^232^

There is a debate in the field whether psychiatric symptoms represent a prodrome or a risk factor for neurodegenerative diseases.^53^ Presence of NPS, especially apathy and depression, predicts MCI conversion to major NCD, specifically in AD and vascular major NCD.^55,131,233-237^ Although an association between mood disorders and risk of major NCD had previously been reported in large epidemiological studies,^235,238^ a recent systematic review and meta-analysis was the first to reveal a significant association of *psychotic disorders* with subsequent development of AD or vascular major NCD.^239^ It is important to note that the association of NPS with major NCDs does not imply causality, and the statistical notion of risk (the probability that an event will occur) is not equivalent to biological vulnerability.^240^ Therefore, it is more appropriate to substitute “probability” for “risk” when we interpret the results of these epidemiological studies.

As discussed above, presence of NPS, particularly delusions, may indicate a high likelihood that the overt stage of the disease will soon follow. The present review summarized a rich body of evidence supporting the idea that NPS are early manifestations of neurodegenerative diseases, particularly when they arise later than the typical onset age for primary psychiatric conditions in the general population.^5,6,146^ The NPS phenomenology also appears to be different in neurodegenerative prodromes, although more research is needed to state this with confidence. For example, sudden onset of severe anxiety around trivial stressors in a previously highly-functioning, well-adjusted individual in their mid-40’s who had never experienced such challenges should prompt consideration of a non-primary psychiatric etiology.^6^ Ghahremani et al. found correlations between levels of a well-validated AD biomarker, plasma phosphorylated tau at threonine 181 (p-tau181) and MBI, although not individual NPS.^241^ Over a 1-year period, MBI was associated with higher p-tau181 levels as well as decline in memory and executive function. These results highlight the significance of MBI as a neurodegenerative disease marker.

It is not entirely clear why NPS occur decades prior to the onset of neurological changes. One hypothesis concerns the selective vulnerability of certain brain areas such as the amygdala, which may be more susceptible to β-amyloid, tau, or α-synuclein deposits.^242,243^ Anatomical mapping studies have shown the amygdala is affected only later in the course of Lewy body spectrum diseases.^244-246^ Nevertheless, subtle changes in brainstem neuronal projections may be present early, leading to limbic circuit dysfunction.^6,121,247-249^ This hypothesis is supported by functional imaging studies performed in patients with prodromal PD.^250,251^

Regarding psychotic symptoms as part of manifest neurodegenerative diseases, major advances in the last decade consist in the clarification of genetic underpinnings, including the discovery of *C9orf72* gene mutations in 2011, now recognized as the most common genetic abnormality associated with bvFTD and familial ALS.^43,44^ This is even more fascinating when juxtaposed with previous studies of bvFTD that did not find psychosis as part of FTD picture.^252^ As detailed in this review, several studies of high quality revealed a heightened risk of psychosis for carriers of *C9orf72* mutations with FTLD spectrum disorders, with some authors reporting increased frequency of delusions early in the course of disease in the FTLD-TDP subtype (often associated with *C9orf72* mutations), compared to other pathological subgroups.^18,43,44,59,80-83^ Better characterization of biomarkers will guide the development of disease-specific diagnostic algorithms and treatment.^155,171^

There is little novelty regarding the management of psychosis of neurodegenerative diseases. No RCTs exist to evaluate nonpharmacological strategies; the lack of evidence may also be due to the fact that behavioral approaches focus on broader NPS, not just psychosis.^167^ Also, psychosis is often associated with agitation, making it difficult to assess distinct study outcomes.^155^ There have been only few RCTs conducted in the past decade to investigate the effectiveness of medications in the treatment of psychosis associated with major NCDs or neurodegenerative diseases, mirroring the dearth of available new psychotropic agents. The exception is pimavanserin, which was met with a great deal of enthusiasm due to its novel mechanism of action. The evidence for pimavanserin has been mixed to date and additional postmarketing data will be helpful to fully elucidate its safety for older adults. Furthermore, there is still very limited evidence pertaining to the management of neurobehavioral aspects of bvFTD and HD, despite the prevalence of severe psychiatric manifestations, including psychosis.

In summary, recent advances in the understanding of psychotic symptoms associated with neurodegenerative diseases center on psychiatric prodromes, including the MBI construct, and their significance as harbingers of full-blown neurobehavioral syndromes. Prompt prodrome recognition is crucial and will allow early intervention with disease-modifying treatments, hopefully in the near future. The symptomatic management of psychosis associated with neurodegenerative diseases, both in the prodromal and manifest stages, remains as much art as it is science, but stronger evidence is needed to support clinical decisions. The complexity of psychotic manifestations warrants management by interprofessional teams that provide coordinated care, integrating nonpharmacological and somatic treatment approaches.
